# Educational intervention regarding diet and physical activity for pregnant women: changes in knowledge and practices among health professionals

**DOI:** 10.1186/s12884-016-0957-1

**Published:** 2016-07-20

**Authors:** Maíra Barreto Malta, Maria Antonieta de Barros Leite Carvalhaes, Monica Yuri Takito, Vera Lucia Pamplona Tonete, Aluísio J D Barros, Cristina Maria Garcia de Lima Parada, Maria Helena D’Aquino Benício

**Affiliations:** Departamento de Nutrição, Faculdade de Saúde Pública, Universidade de São Paulo - USP, Av. Dr. Arnaldo, 715, São Paulo, São Paulo 01246-904 Brazil; Rua Egidio Martins, 160 ap 315 Ponta da Praia, Santos, São Paulo 11030160 Brazil; Departamento de Enfermagem, Faculdade de Medicina de Botucatu, Universidade Estadual Paulista - UNESP, Av. Professor Montenegro, 18618970 Botucatu, São Paulo Brazil; Departamento de Pedagogia do Movimento do Corpo Humano, Escola de Educação Física e Esporte, Universidade de São Paulo - USP, Av. Prof. Mello Moraes, 65, 05508-030 São Paulo, São Paulo Brazil; Departamento de Medicina Social, Faculdade de Medicina, Universidade Federal de Pelotas, Rua Mal. Deodoro, 1160, 3ºpiso, Pelotas, 96020220 Rio Grande do Sul Brazil

**Keywords:** Attitudes and Practices in health, Antenatal care, Advice

## Abstract

**Background:**

The knowledge and practices of health professionals have a recognized role in behaviors related to the health of their patients. During pregnancy, this influence can be even stronger because there is frequent contact between women and doctors/nurses at periodic antenatal visits. When trained, supported and motivated, these professionals can act as health promoters. This study aimed to evaluate the effect of a focused educational intervention on improving the knowledge and practices of health professionals concerning diet and physical activity during pregnancy.

**Methods:**

A controlled, non-randomized study was performed to assess the effects of an educational intervention on the knowledge and practices of nurses and doctors who provide primary care to pregnant women. The intervention group, doctors and nurses (*n* = 22) from the family health units in a medium-sized city of São Paulo State, Brazil, received 16 h of training comprising an introductory course and three workshops, whereas the control group, doctors and nurses (*n* = 20) from traditional basic health units in Botucatu, did not. The professionals’ knowledge was assessed at two time points, 1 month prior to and 1 year after the beginning of the intervention, using an ad hoc self-report questionnaire. The increases in the knowledge scores for walking and healthy eating of the intervention and control groups were calculated and compared using Student’s t-test. To analyze the professionals’ practice, women in the second trimester of pregnancy were asked whether they received guidance on healthy eating and leisure-time walking; 140 of these women were cared for by professionals in the intervention group, and 141 were cared for by professionals in the control group. The percentage of pregnant women in each group that received guidance was compared using the chi-square test and the Prevalence Ratio (PR), and the corresponding 95 % confidence intervals (CI) were calculated.

**Results:**

The intervention improved the professionals’ knowledge regarding leisure-time walking (92 % increase in the score, *p* < 0.001). The women who were cared for by the intervention group were more likely to receive guidance regarding leisure-time walking (PR = 2.65; 95 % CI = 1.82-3.83) and healthy eating (PR = 1.76; 95 % CI = 1.34-2.31) when compared to the control group.

**Conclusion:**

It is possible to improve the knowledge and practices of health professionals through the proposed intervention aimed at primary health care teams providing antenatal care.

## Background

Behaviors such as maintaining a sedentary lifestyle [1, 2] and unhealthy dietary habits [3, 4] are prevalent among pregnant women worldwide. These public health problems are associated with excessive weight gain [5, 6], low birth weight [7], prematurity [8, 9], cesarean delivery [10], gestational diabetes [11] and pregnancy-induced hypertension [12].

The evidence indicating the influence of diet and physical activity during pregnancy on mother-child outcomes is increasingly gaining the attention of researchers and public health managers because, among other reasons, these factors are potentially modifiable [13]. Several factors might influence eating behaviors and physical activity, including the guidance and support provided by healthcare professionals [13–15]. A recent systematic literature review suggested that nutritional counseling effectively increases adherence to healthy eating patterns; nevertheless, the same review showed that professionals do not routinely promote such practices [16]. Similarly, some researchers have emphasized that the guidance provided by healthcare professionals increases physical activity among pregnant women [17].

A study conducted in the United States found that the desire to exercise during pregnancy was higher among women cared for by obstetricians who discussed physical activity during antenatal visits [18]. Similarly, a study in Finland showed that physical activity counseling during antenatal visits effectively sustained exercise levels among pregnant women [19].

In antenatal care, there are some barriers that hinder the promotion of physical activity and healthy eating; these include lack of time and lack of incentives [20]. Doctors’ and nurses’ lack of knowledge of current recommendations in these areas is one of the barriers that can be modified by educational intervention. Previous studies have indicated that many professionals who provide care to pregnant women are not acquainted with the current guidelines for physical activity during pregnancy and have limited knowledge regarding its benefits and recommendations for safe practice [21–23]. In addition, a recent literature review showed that healthcare professionals in developed countries rarely discuss issues such as nutrition during pregnancy with their patients; moreover, they consider their lack of training to be a hindrance in this regard [16].

During pregnancy, women maintain frequent contact with doctors and nurses during periodic antenatal visits. Therefore, these professionals, as suppliers of knowledge and support, might play a relevant role in the promotion of behavioral changes [24, 25]. Pregnant women become strongly motivated to change their behaviors when they are made aware of the positive effects that healthy eating and physical activity have on pregnancy outcomes [19, 26].

Health promotion programs focused on physical activity and healthy eating are increasingly encouraged and developed in Brazil; the primary targets of such programs are adults [27, 28]. Certain health ministry-supported initiatives aim to train healthcare professionals and to formulate strategies to include the active promotion of physical activity and healthy eating within the primary care system [29]. However, no study has assessed educational interventions aimed at training professionals who provide antenatal care in Brazil to systematically promote physical activity and guide healthy eating among pregnant women. The present study aimed to evaluate the effect of a focused educational intervention on improving the knowledge and practices of health professionals with regard to diet and physical activity during pregnancy.

## Methods

### Design

A controlled, non-randomized study targeting pregnant women cared for at primary health care facilities was conducted in the city of Botucatu, state of São Paulo, Brazil from 2012 to 2014. Botucatu is a municipality located in the Brazilian state with the highest socioeconomic level; it is 250 km from the state capital, São Paulo city, and has approximately 130,000 inhabitants, 93 % of whom reside in the urban area [30].

The study compared the knowledge and practices of two groups of healthcare professionals. The intervention group initially consisted of 23 doctors and nurses who participated in a 16-h intervention package that included an introductory course and three workshops. The control group (*n* = 20) was composed of doctors and nurses who did not receive any intervention. The intervention group comprised staff from nine family health units, and the intervention group included staff from eight traditional primary care units. The Family Health Strategy introduced in Brazil in 1994 is based on a novel concept of a health team including a doctor, nurses and four community health workers who together provide medical care to approximately 1,000 families living in a well-defined geographic area. Traditional health care units in Brazil are not geographically defined and do not include community health workers. More details concerning the Family Health Strategy can be found elsewhere [31].

Based on the methodological framework used to assess collective health interventions [32], the present article describes the evaluation of the intervention process as a basis for later analysis of the effect of the intervention on the target population.

### Population and sample

All of the doctors and nurses (*n* = 43) who provided low-risk antenatal care within the public health network of the urban area of Botucatu municipality were invited to participate in this study; none refused. One of the participants was excluded because he was disaffiliated from the municipal primary care staff during the study. Therefore, the intervention group was composed of 22 participants, and the control group was composed of 20 participants.

Group allocation was not randomized. The choice to include only professionals from family health units in the intervention group and only professionals from traditional basic health units in the control group aimed to align the study with the current primary care policy in Brazil, which prioritizes the Family Health Strategy [33]. In addition, this choice acknowledged that it would not have been possible to prevent the professionals from the family health units from meeting and sharing the content of the intervention because these individuals are required to attend the monthly administrative and technical/scientific meetings held by the municipality’s family health unit primary care managers.

To analyze the professionals’ practice, women in the second trimester of pregnancy were interviewed; 140 of these women were cared for by professionals from the intervention group, and 141 were cared for by the control group. The sample size provided 95 % power and a 5 % significance level to detect differences in the percentage of women who received guidance on healthy eating and leisure-time walking.

To be included in the study, pregnant women were required to be enrolled in the low-risk antenatal care program of the Botucatu public healthcare network, be 18 years of age or older and have started antenatal care beginning at gestation week 13. Of the 353 women in the second trimester of pregnancy who were selected for the present study, 38 of those allocated to the intervention group and 27 of those allocated to the control group were not eligible. One woman allocated to the intervention group could not be located, and six (1.7 %, two in the intervention group and four in the control group) refused to participate.

### Intervention

In the context of the Continuing Health Education program [34], an educational intervention was developed to improve doctors’ and nurses’ knowledge concerning healthy eating and physical activity during pregnancy and to systematically promote healthy eating and leisure-time walking as part of the provided antenatal care.

The results of a previous cross-sectional study concerning the physical activity of low-risk pregnant women who received care from primary care units in the same city were considered in the design of the educational intervention [2], as were the results of a qualitative study that identified barriers to and facilitators of healthy eating and physical activity among pregnant women from Botucatu [35]. The professionals’ knowledge and practices regarding their patients’ physical activity and healthy eating during pregnancy prior to intervention were also considered.

International guidelines provided the technical/scientific framework used to define the recommendations for walking during pregnancy (i.e., 30–40 min of walking at moderate intensity five or more times per week) [36, 37]. The healthy eating guidelines were based on the Brazilian recommendations for pregnant women [38]: three fruits; two portions of vegetables (one raw and one cooked); two portions of beans (one at lunch and one at dinner, at least 5 days per week); and restriction of soft drinks and industrially processed cookies (once per week at most).

The intervention lasted 8 months and comprised an introductory course and three workshops. The introductory eight-hour course recommended physical activity and healthy eating throughout pregnancy and provided the scientific basis for this recommendation. Furthermore, it updated the recommendations for weight gain during pregnancy as well as the status of pregnant women with regard to physical activity, healthy eating and their determinants, described the stages of behavioral change according to the Transtheoretical Model [39], used a motivational interview to improve the professionals’ interactions with pregnant women [40] and acknowledged the barriers to and facilitators of the active promotion of leisure-time walking and healthy eating during antenatal care. Throughout the course, the professionals designed the initial version of a plan to promote leisure-time walking and healthy eating as part of the antenatal care provided at the family health units. Finally, the participants completed a questionnaire to evaluate the course.

The workshops were conducted at each family health unit with the entire staff. The first workshop (4 h) involved designing a plan for systematizing the promotion of leisure-time walking and healthy eating within the local antenatal care routine. On this occasion, the nurses and doctors agreed that they would provide counseling with regard to healthy eating, leisure-time walking and appropriate weight gain during pregnancy in all their consultations; moreover, they would record the actions taken. Materials were printed to support counseling, including a form to guide and record the assessment of weight gain, a form to guide the provision of advice with regard to walking and nutrition, a tutorial that explained how to complete the form and folders to distribute to the pregnant women that described the recommendations for leisure-time walking and healthy eating as well as their benefits. The printed materials were delivered to the participants during the workshop.

The second and third workshops each involved two hours of activity. The purpose of the second workshop was to evaluate the effects of the first workshop, survey the difficulties that the professionals had met and present proposals for promoting implementation of leisure-time walking and healthy eating in their antenatal care. The third workshop sought to reinforce knowledge, agree upon procedures, reevaluate the difficulties met and offer ways to overcome them. New support materials were delivered on that occasion, including a summary form listing all of the selected healthy eating habits and the recommendations for walking during pregnancy and two banners containing a synthesis of the messages (healthy eating and walking during pregnancy) to be placed in the common areas of the family health units. The purpose of providing these materials was to facilitate the professionals’ task in antenatal care visits by providing visual and practical support for such recommendations to pregnant women.

### Data collection

The knowledge of the professionals allocated to the intervention and control groups was assessed at two time points, 1 month before and one year after the introductory course, using an ad hoc self-report questionnaire. Researchers with previous experience in assessing the knowledge of healthcare professionals specifically designed the questionnaire for the present study. The questionnaire was pilot tested, adapted and retested until the final 14-question version was created. Nine questions assessed the professionals’ knowledge of the current recommendations for leisure-time walking (i.e., its recommended frequency, duration and intensity in each trimester). The other five questions assessed the professionals’ knowledge of the dietary recommendations for pregnant women regarding the frequency of intake and recommended portions of fruit, vegetables, beans, soft drinks and industrially processed cookies.

To assess the inclusion of practical guidance with regard to walking and healthy eating in the consultations, the pregnant women cared for by the two groups of professionals were asked prior to the administration of the questionnaire whether they had been given such guidance during the antenatal care visits performed during their second trimester. This information was collected after adding specific respond-at-home questions to the survey for future assessment of the effect of the intervention on the women’s behaviors.

To evaluate the introductory course, the professionals responded to an ad hoc questionnaire that assessed their perception of the course content, the consistency between the topics addressed and their practical experience in family health units.

### Demographics

The following variables were used to characterize the sample: group (intervention or control); age (in years); profession (nurse or doctor); specialization course; latu-sensu graduate program (yes, no); duration of work in the current healthcare unit (in years); and duration of work in antenatal care (less than 5 years, 5–10 years, or more than 10 years).

The following variables were used to evaluate the professionals’ knowledge and practices: knowledge score for walking before and after the educational intervention (1 point was assigned for each correct answer to the questions on recommended frequency, time and intensity of leisure-time walking per trimester; the total score ranged from 0 to 9); knowledge score for healthy eating before and after the educational intervention (1 point was assigned for each correct answer to questions regarding the recommendations for fruit, vegetable, bean, soft drink and cookie intake; the total score ranged from 0 to 5); increase in the knowledge score for walking (score for walking after – score for walking before); increase in the knowledge score for healthy eating (score for healthy eating after – score for healthy eating before); whether pregnant women received guidance with regard to leisure-time walking during pregnancy (yes or no); and whether pregnant women received guidance with regard to healthy eating during pregnancy (yes or no).

The variables used to assess the introductory course were the topics addressed (excellent, good or fair/poor) as well as the consistency between the topics addressed and the actual practices at the family health unit (excellent, good or fair/poor).

### Statistical analyses

The data were entered twice at the time of collection using Microsoft Office Access. After checking the data for accuracy and correcting errors, the dataset was transferred to Stata version 13.0, which was used to assess the consistency of the data and to perform statistical analyses. We adopted alpha <0.05 as the statistical significance level.

The characteristics of the intervention and control groups were compared using Student’s t-test or the chi-square test according to the nature of each variable. For the intervention group, the frequencies of the professionals who rated the topics addressed in the introductory course and their consistency with the actual practices performed at the family health units as excellent, good, or fair/poor were calculated.

The means and standard deviations (SD) of the professionals’ knowledge scores (walking and healthy eating), before and after the educational intervention, were calculated. The between-group differences in the scores obtained prior to the educational intervention were analyzed using Student’s t-test. Because the knowledge of the professionals in the two groups did not differ prior to the intervention, the increases in the knowledge scores for walking and for healthy eating were calculated separately. The average increases in the intervention group and in the control group were compared using Student’s t-test. Some variables (age, duration of work at the healthcare unit) showed significant between-group differences; however, additional analysis of the initial knowledge scores did not confirm them as potential confounders. The possible differences between doctors and nurses in knowledge prior to educational intervention with regard to leisure-time walking and healthy eating were evaluated using Student’s t-test.

The percentage of pregnant women who reported receiving guidance with regard to leisure-time walking and healthy eating from both groups of professionals was compared, and the difference was assessed using the chi-square test. In addition, the Prevalence Ratio (PR) and corresponding 95 % confidence intervals (CI) were calculated.

## Results

All of the doctors and nurses who provided low-risk antenatal care within the public health network of the urban area of Botucatu municipality participated in this study. There were 20 professionals in the control group and 22 in the intervention group.

A significant between-group difference with regard to profession was not found (*p* = 0.889); however, there were more doctors in the intervention group than in the control group (54 % vs. 40 %). The number of professionals with more than 10 years of experience in antenatal care was approximately three times higher in the control group than in the intervention group; however, this difference was not significant (*p =* 0.15). The average age of the professionals in the intervention group was 34.2 (SD = 8.0) years, whereas the average age of those in the control group was 39.4 (SD = 10.2) years. The average duration of work in the current healthcare unit was 2.0 (SD = 1.7) years in the intervention group and 8.2 (SD = 8.9) years in the control group. These differences were significant (Table 1).

**Table 1:** Professional characteristics of the study groups

Characteristics	Intervention (*n* = 22)		Control (*n* = 20)		
	Mean	(SD)	Mean	(SD)	*P-value*
Age (years)	34.2	(8.0)	39.4	(10.2)	0.041
Duration of work in unit (years)	2.0	(1.7)	8.2	(8.9)	0.003
	*N*	(%)	*N*	(%)	
Profession					
Doctors	12	(54.6)	08	(40.0)	0.889
Nurses	10	(45.4)	12	(60.0)	
Specialization (course)	20	(90.9)	17	(85.0)	0.349
Duration of work in antenatal care (years)					
Less than 5	10	(45.5)	06	(30.0)	0.151
5 to 10	09	(40.9)	06	(30.0)	
More than 10	03	(13.6)	08	(40.0)	

The professionals were satisfied with the topics addressed in the introductory course, given that 79.3 % rated them as excellent and 20.7 % as good. All of the participating professionals considered the topics to be consistent with the actual practices performed at family health units.

There were no differences in the mean knowledge scores of doctors and nurses prior to educational intervention: leisure-time walking, *p* = 0.1158 (doctors 4:25 (SD = 0:50); nurses 3:22 (SD = 1.88)), and healthy eating, *p* = 0.2547 (doctors 3.25 (SD = 0.97); nurses 3.63 (SD = 1.18). Thus, the following results were obtained by considering doctors and nurses as a single group (health professionals).

The knowledge scores obtained prior to the educational intervention did not differ between the groups. The average knowledge score for leisure-time walking was 3.8 (SD = 2.5) in the intervention group and 3.6 (SD = 1.7) in the control group (*p* = 0.89). The average knowledge score for healthy eating was 3.6 (SD = 1.0) in the intervention group and 3.4 (SD = 1.2) in the control group (*p* = 0.63).

**Fig. 1:**
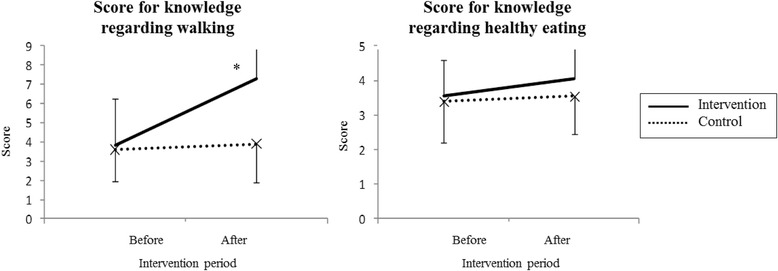
Average knowledge scores for walking and healthy eating during pregnancy before and after educational intervention

The knowledge scores of the intervention and control groups after the educational intervention were 7.3 (SD = 1.7) and 3.9 (SD = 2.0), respectively, for walking and 3.9 (SD = 2.0) and 3.6 (SD = 1.1), respectively, for healthy eating (Fig. 1).

The average increase in the knowledge score regarding walking was 3.5 for the intervention group and 0.3 for the control group, a significant difference (*p* = 0.0001). The average increase in the knowledge score regarding healthy eating showed no significant difference between the groups (intervention = 0.55; control = 0.15; *p* = 0.1296).

Table 2 shows that women were more likely to receive guidance regarding leisure-time walking and healthy eating at an antenatal care visit during their second trimester when they were cared for by professionals in the intervention group than when they were cared for by professionals in the control group (leisure-time walking: I = 50.7 %; C = 19.1 %; healthy eating: I = 58.6 %; C = 33.3 %).

**Table 2:** Number and percentage of pregnant women who received guidance regarding leisure-time walking and healthy eating (the values shown are number (%))

Guidance received	Intervention (*n* = 140)	Control (*n* = 141)	PR (95 % CI)	*p*
Leisure-time walking				
Yes	71 (50.7 %)	27 (19.1 %)	2.65 (1.82–3.83)	<0.001
No	69 (49.3 %)	114 (80.9 %)	1	
Healthy eating				
Yes	82 (58.6 %)	47 (33.3 %)	1.76 (1.34–2.31)	<0.001
No	58 (41.4 %)	94 (66.7 %)	1	

## Discussion

The present study assessed an intervention undertaken with primary care nurses and doctors seeking to improve the knowledge and motivation needed to actively promote walking and five healthy eating practices among pregnant women at antenatal care visits. The results were positive in terms of improving the professionals’ knowledge regarding the current recommendations for leisure-time walking during pregnancy. In addition, the professionals in the intervention group provided guidance regarding leisure-time walking and the five healthy eating practices more frequently than those in the control group.

Given that the participants were not randomly allocated to the intervention or control groups in the present study, some differences between intervention and control groups were expected. The professionals in the intervention group were younger and had worked at health care units for less time; however, those variables were found not to be confounders.

One additional factor deserving of consideration is the possible induction effect resulting from the completion of questionnaires by the participants prior to the intervention. This action might have favorably influenced the results of the second assessment by raising the awareness of the professionals with regard to the investigated topics, thereby leading them to seek information, discuss the topics more frequently in their consultations with pregnant women, or both. Because increased knowledge of the topics addressed based on the second questionnaire was not observed in the control group, it may be inferred that the results were due to the educational intervention.

The validity of the results is also supported by the procedure used to assess the between-group differences regarding the actual guidance provided to pregnant women. Traditionally, assessment of this practice is based on professionals’ reports or direct observation during consultations; the latter is considered a superior approach because individuals tend to overestimate the frequency with which they perform desirable procedures or actions, thereby resulting in information bias [41]. The use of pregnant women to assess the frequency with which professionals provided guidance with regard to leisure-time walking and healthy eating during antenatal care visits was a suitable third option because it avoided the need to perform laborious and expensive observations and avoided the errors that result from the use of self-reports. In addition, the use of pregnant women enables one to assume that the quality of the guidance provided by the professionals was evaluated because the women likely only remembered and reported significant encounters with their healthcare providers but forgot more superficial encounters [42].

The professionals in both groups were expected to have more knowledge of the dietary recommendations than of the physical activity recommendations during pregnancy because the literature on healthy eating during pregnancy is older and more extensive [43–45], because dietary assessments and the promotion of healthy eating have been included as part of antenatal care visits in Brazil for more than 20 years [29], or because of both. This hypothesis was confirmed by the results and might account for the lack of effect of the intervention on the healthy eating knowledge scores. The lack of change regarding the healthy eating knowledge scores notwithstanding, women who were cared for by professionals in the intervention group were more likely to report receiving guidance on healthy eating than those cared for by professionals in the control group. This finding indicates the positive effect of the intervention. In addition to updating their knowledge, importantly, the educational intervention aimed to motivate the professionals, engage them and facilitate their work so that they would effectively promote healthy eating within their antenatal care routine, thereby making that result a reality.

The positive results regarding the inclusion of healthy eating and walking guidance in the consultations might have resulted from better understanding of the change process associated with the health-related behaviors of the professionals and the improved communication between the professionals and the pregnant women. Importantly, the stages of behavioral change described in the Transtheoretical Model [39] and the motivational interviewing technique [40] were addressed in the educational intervention. Moreover, the distribution of printed materials was specifically tailored to facilitate the selection of the most relevant guidance to be provided at each consultation. Together, the elements included in the educational intervention might have helped improve the communication between the professionals and their pregnant patients [42], thereby significantly increasing the percentage of women who had an opportunity to discuss healthy eating and walking at their antenatal care visits.

Importantly, few of the professionals (doctors and nurses, with no difference between them) in either group were acquainted with many of the current recommendations for physical activity during pregnancy, especially those for leisure-time walking, prior to the intervention. This situation was favorably modified in the intervention group. The results of a study conducted in Michigan, USA, were similar: 73 % of investigated obstetricians were not familiar with the guidelines of the American College of Obstetricians and Gynecologists (ACOG) for exercise and pregnancy [21]. Another study performed in Brazil found that few physicians (7.9 %), nurses (9.1 %) or community healthcare workers (3.6 %) knew the current recommendations for physical activity during pregnancy [23]. A survey study conducted in South Africa showed that a large majority (83 %) of the doctors who responded were not familiar with the ACOG guidelines for exercise during pregnancy [46]. Thus, the need for continuing health education action targeting this subject is obvious.

The unfavorable scenario observed regarding the professionals’ knowledge of physical activity during pregnancy results in low encouragement of pregnant women to begin or maintain regular physical activity. This scenario might be explained given that past recommendations indicated the possible negative effects of excessive physical effort during pregnancy [47]. However, the current evidence shows that exercise of appropriate frequency, duration, and intensity protects maternal and fetal health and is associated with favorable perinatal outcomes [6, 8, 36, 48].

Researchers who investigate the process of healthcare service innovation dissemination argue that the exposure of individuals to new knowledge has little effect when the knowledge is not perceived as relevant to the healthcare facility or the individual. For this realization to occur, new knowledge or practices must be perceived as somehow better (i.e., saving time, leading to patient improvement, increasing the professional’s or the service’s prestige, and so on). In addition, novelties should be compatible with the professional’s needs and values, simple and result in favorable and easily perceived consequences [49]. We take this into account in our intervention.

In summary, the educational intervention used in this study promoted changes in the knowledge and practices of the professionals in the family health units. Furthermore, it has the potential to affect the behaviors of the pregnant women for whom these professionals care; this outcome will be assessed in the larger research project of which the present study is a part. There is, though, space for improving the intervention given that a large proportion of the women in the intervention group did not receive suitable guidance.

## Conclusions

The current intervention was effective, and it accomplished all of the intended goals except that of increasing professionals’ knowledge regarding the dietary recommendations for pregnant women. Only the intervention group exhibited significant increases with regard to the knowledge score for leisure-time walking. The women most likely to receive guidance regarding walking and healthy eating during their prenatal care visits were those cared for by the professionals in the intervention group. This finding might be attributable to the educational intervention.

## Abbreviations

CI, Confidence interval; PR, Prevalence ratio; SD, Standard deviation
